# Medicine storage, wastage, and associated determinants among urban households: a systematic review and meta-analysis of household surveys

**DOI:** 10.1186/s12889-021-11100-4

**Published:** 2021-06-12

**Authors:** Adineh Jafarzadeh, Alireza Mahboub-Ahari, Moslem Najafi, Mahmood Yousefi, Koustuv Dalal

**Affiliations:** 1 Department of Health Economics, School of Management and Medical Informatics, Tabriz University of Medical Sciences, Tabriz, Iran; 2 Department of Health Economics, Iranian Evidence-Based Medicine Center of Excellence, Tabriz University of Medical Sciences, Tabriz, Iran; 3 School of Management and Medical Informatics, Daneshgah Street, Daneshgah Square, Tabriz, Iran; 4 Department of Pharmacology &Toxicology, Faculty of Pharmacy, Tabriz University of Medical Sciences, Tabriz, Iran; 5 Iranian Center of Excellence in Health Management, Department of Health Economics, School of Management and Medical Informatics, Tabriz University of Medical Sciences, Tabriz, Iran; 6 Division of Public Health Science, Institute of Health Sciences, Mid Sweden University, Sundsvall, Sweden; 7 Department of Epidemiology, Biostatistics and EBM, Faculty of Medicine and Health Care, al-Farabi Kazakh National University, Almaty, Kazakhstan

**Keywords:** Medicine, Home storage, Wastage, Household, Not in use, Expired

## Abstract

**Background:**

Irrational household storage of medicines is a world-wide problem, which triggers medicine wastage as well as its associated harms. This study aimed to include all available evidences from literature to perform a focused examination of the prevalence and factors associated with medicine storage and wastage among urban households. This systematic review and meta-analysis mapped the existing literature on the burden, outcomes, and affective socio-economic factors of medicine storage among urban households. In addition, this study estimated pooled effect sizes for storage and wastage rates.

**Methods:**

Household surveys evaluating modality, size, costs, and affective factors of medicines storage at home were searched in PubMed, EMBASE, OVID, SCOPUS, ProQuest, and Google scholar databases in 2019. Random effect meta-analysis and subgroup analysis were used to pool effect sizes for medicine storage and wastage prevalence among different geographical regions.

**Results:**

From the 2604 initial records, 20 studies were selected for systematic review and 16 articles were selected for meta-analysis. An overall pooled-prevalence of medicine storage and real wastage rate was 77 and 15%, respectively. In this regard, some significant differences were observed between geographical regions. Southwest Asia region had the highest storage and wastage rates. The most common classes of medicines found in households belonged to the Infective agents for systemic (17.4%) and the Nervous system (16.4%). Moreover, income, education, age, the presence of chronic illness, female gender, and insurance coverage were found to be associated with higher home storage. The most commonly used method of disposal was throwing them in the garbage.

**Conclusions:**

Factors beyond medical needs were also found to be associated with medicine storage, which urges effective strategies in the supply and demand side of the medicine consumption chain. The first necessary step to mitigate home storage is establishing an adequate legislation and strict enforcement of regulations on dispensing, prescription, and marketing of medicines. Patient’s pressure on excessive prescription, irrational storage, and use of medicines deserve efficient community-centered programs, in order to increase awareness on these issues. So, hazardous consequences of inappropriate disposal should be mitigated by different take back programs, particularly in low and middle income countries.

**Supplementary Information:**

The online version contains supplementary material available at 10.1186/s12889-021-11100-4.

## Background

Home storage of medicine is a public health problem worldwide, which occurs because of improper utilization of medicines and/or non-adherence with drug therapy that consequently affects health, environment, and healthcare services [1–6]. Although timely access to medicines is essential, there are some global concerns regarding unnecessary storage and inappropriate usage of medicines as well as unsafe disposal of leftover medicines [7–11]. During past years, medicine utilization pattern has changed, which consequently leads to greater purchasing volumes and excessive accumulation at home [12]. According to the World Health Organization (WHO), more than 50% of medicines are inappropriately prescribed and dispensed, which causes unnecessary storage [13]. Therefore, WHO (2011) calculated that about 24.9% of the total health expenditure or 1.4 to 1.63% of Gross Domestic Product worldwide were spent on medicines [14]. In Asia, a large proportion of out of pocket payments is spent on medicines. For example, in India, Bangladesh, and Vietnam, this share is calculated as 70% [15]. In addition, households in Saudi Arabia spent a total of US $150 million on medicines that were never consumed [16]. Garg et al. in their study has reported that three-fourth of out-of-pocket expenditures (74% rural and 67% urban) is drug-related [17]. In China, the share of medicine expenses from GDP has raised more than up to 320 times since 1980 [18]. Such observations only indicated the immediate consequences of inappropriate supply and demand of medicines, whereas further concerns would be attached to the risk of inappropriate storage and disposal. For example, accidental intoxication, inappropriate self-medication, the presence of pharmaceutical ingredients in waterways as environmental pollutants, accidental poisoning of wildlife, and risk of antibacterial resistance can be named in this regard [8–10, 19–21].

By reviewing the medicine utilization chain, it should be clarified that unpleasant outcomes to both individuals and societies arise from several sources. Accordingly, it usually starts with over prescription by physicians [9, 22, 23]; then continues through making improper decisions such as over-purchasing [12], non-adherence to treatment [16, 23, 24], and saving medicines for future use [3, 25, 26]; and finally lasts with inappropriate disposal [27–31] . Several systematic reviews have been conducted to address the issue; for example, on self-medication practice [32–38], medicine wastage [7, 39], and disposal practices among households [40, 41]. However, to the best of our knowledge, there were inadequate consensuses about global burden of medicine storage rate, modality, and the associated factors. Of note, only one systematic review was found investigating this issue, which studied medicine storage from 1999 to 2016 [42]. The Authors included studies from different population groups (consisting of university students, hospital referrals, and urban residents), which according to our electronic search, 8 and 7 studies were found to be performed before and after 2016, so we believed that they are eligible to support this body of literature. Another important issue regarding this review is that because of the existing heterogeneity in sample population of the included studies, the authors were not able to estimate the global or regional prevalence of inappropriate storage, so they narratively reported medicine storage rate by drug names and types. Correspondingly, this type of reporting would subsequently limit the comparability of findings from different settings or regions [43]. Our study was conducted to overcome the above-mentioned challenges through performing a comprehensive overview of recent literature. Three main contributions of this review could be highlighted as follows: First, we investigated further 15 household surveys that have been published before and since the last search date of Hussein’s review. Second, in this study, we estimated the global and regional burden of medicine storage and wastage. Third, in order to increase the international and regional comparability of medicine utilization findings, we reported the obtained results based on a standard methodology called ATC classification [44].

This study aimed to include all available evidences obtained from literature for performing a focused examination of the prevalence and the associated factors affecting medicine storage among households. It is noteworthy that, medicine storage is a public health problem in both rural and urban areas; however, the prevalence of home storage was reportedly higher in urban households due to more facilities concentrated within these areas as well as easier access to medicines [2, 45]. Therefore, due to this reason, we included household surveys that investigated and reported information about urban populations.

## Method

This systematic review and meta-analysis was conducted and reported in terms of the Preferred Reporting Items for Systematic Reviews and Meta-Analyses (PRISMA) statements [46] (Additional file 1).

### Eligibility criteria

Studies were included in this systematic review if they were household surveys that:

1. Investigated medicine storage among households through face to face interview and then reported data for urban households.
2. Reported rates of the wasted medicines as a percentage of household’s overall storage (real wastage, potential or both)
3. Were written in English.

Studies were excluded if they:

1. Had mixed samples of households without clearly defining independent data for urban households.
2. Reported medicine wastage ratio as a proportion of households, rather than medicine type.
3. focused on a specific medicine (e.g. antibiotics) or a population subgroup (patients, elderly or children).
4. Investigated medicine wastage without addressing or reporting overall medicine storage.
5. Were editorials, commentaries, and opinion pieces.

### Search sources and search strategies

PubMed, EMBASE, OVID, SCOPUS, ProQuest, and Google scholar databases were searched up to February 2016. All of the keywords were in English and the search strategy was restricted to English language publications. The terms used in this review were informed using a recently performed systematic review [7] and then expanded after co-author agreement (Additional file 2). The electronic search was complemented by hand-searching of the related articles as well as the reference lists of the final studies. The research update was conducted using the same strategy in February 2019.

### Screening and study selection

Search results were imported and managed via EndNote X8 (Thomson Reuters, New York, USA). Duplicates were firstly removed electronically and then manually. Subsequently, the title and abstract of the included studies were independently screened by two reviewers (AM and AJ), and disagreements were finally resolved by helping a third reviewer (MN). Full-text of potential studies were retrieved and reviewed by the two reviewers. In order to obtain inaccessible full-texts or English version of the included papers, email or ResearchGate contact was made by the authors.

### Data extraction

Two reviewers (AM and AJ) extracted data for the country where the study was conducted, year of publication, sample size, recall period, medicine storage rate (as a percentage of households), medicine per household, real medicine wastage (expired or with no expiration date), potential medicine wastage (not in used medicine), category of medicines, category of wastage medicine, the frequency of individual medicine, factors associated with storage and wastage medicines, total cost of medicines, dosage forms of medicine, storage place, and disposal method. To compare cost data across different settings, the calculated costs were converted into United States (US) dollars ($). For studies that did not report their findings based on ATC classification, we classified the individual medicine or any reported category in terms of the Guidelines for ATC Classification and Daily Dose Defined (DDD) assignment (2018) [44].

### Risk of bias assessment

The methodological quality of the eligible studies was assessed using an adaptation of the quality assessment checklist for household surveys, which was developed and used by West et al. comprising 17 items [7]. Each question scored 2, 1, and 0 if they were answered as yes, partial, and unclear or no. The total score for the study was calculated using the following formula: [(number of “yes” * 2) + (number of “partials” * 1)]/ [34 – (number of “N/A” * 2)]. All the included studies were ranked according to the total score from 17 items, then the overall risk of bias was classified as high, moderate, and low if a study got ≤ 50%, 50–75%, and ≥ 75% of the total score, respectively.

### Data analysis

Meta-analysis was conducted in order to synthesize the prevalence of both medicine storage and wastage using random-effects model by the sample size weighting. The results were presented with 95% confidence intervals (95% CI) [47]. Statistical heterogeneity among the studies were assessed by Cochran’s Q statistic and I^2^ index [48, 49]. As the analytical results revealed a high heterogeneity, the random-effects model was employed. All these statistical analyses were conducted using the STATA software, version 16 (Stata Corp. LP, College Station, TX, USA). As well, predefined subgroup analyses were conducted by geographical region. In order to report mean cost of medicine storage and wastage, all the reported costs were adjusted for inflation using the Consumer Price Index (CPI) for original countries, and converted to 2011 international dollars using the Purchasing Power Parity (PPP) conversion factor.

## Results

**Fig. 1:**
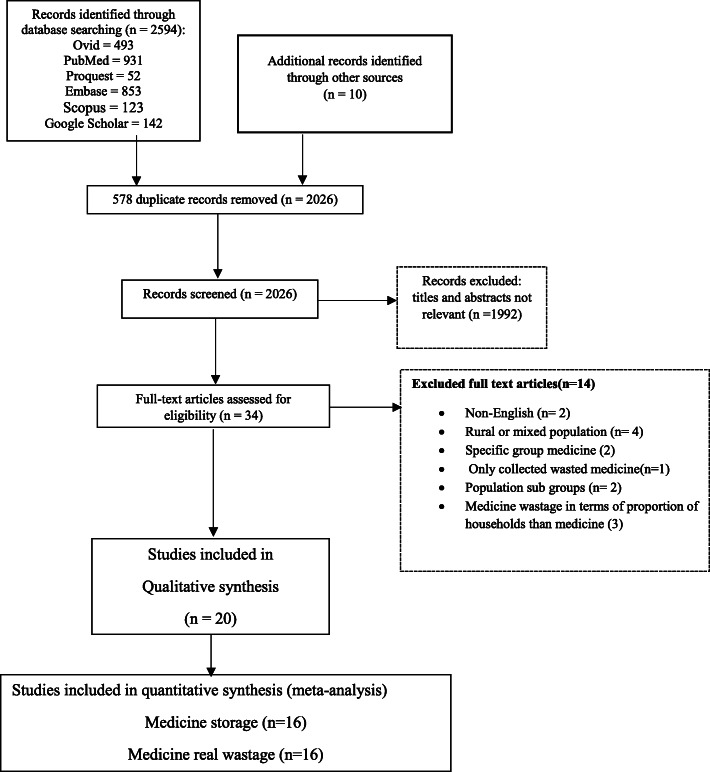
PRISMA Chart describing systematic review search process

From a total of 2604 initially identified citations, 578 were duplicates and 1992 did not meet the inclusion criteria of this study. Finally, 34 full texts were completely scrutinized, of which 14 were excluded because of the lack of relevance, and 20 articles were recognized as eligible for the final review. A study was excluded because it was conducted in a rural area [50]. Although three article papers studied both urban and rural populations, we were not able to separate results for urban subjects [2, 24, 51]. Wongpoowarak et al. in their study reported only medicine wastage without providing data for storage modalities and its determinants [52]. Additionally, three studies were excluded because they reported medicine storage in terms of the household, rather than medicine [30, 53, 54]. Two studies were non-English [55, 56], 2 studies addressed a specific medicine [57, 58], and 2 studies were performed on a particular population subgroup [59, 60]. Figure 1 illustrates a summary of the literature identified at each one of the stages of the process.

### Characteristics of the included studies

All the studies were cross-sectional, which used a questionnaire for gathering the relevant data from the study subjects. Two studies investigated both urban and rural household to assess medicine wastage, but only urban household data were extracted [28, 61]. A total of 10,898 households were included across the 20 studies conducted between 2002 and 2017. The majority of the studies were published from 2010 onwards (*n* = 16, 80%). The sample sizes varied within the studies included in this review. The lowest sample size was 97 households, whilst the highest sample size was 2600 (Table 1). Of the 20 included studies, 16 studies were from Asia and Africa, 3 from Europe, and 1 from South America.

**Table 1:** Extracted study characteristics

Author, year	Country	Sample size	Recall Period (Month)	prevalence of medicine storage (%, household)	Medicines per Household, Mean [SD]	Potential Wastage (%, medicine)	Real Wastage (%, medicine)
Abou-Auda 2002, [16]	Saudi Arabia	1554	In 2001	99.67	8 [4.3]	4.26	21.54
Abou-Auda 2002, [16]	Persian Gulf Countries	87	In 2001	–	7.1	3.3	38
Abushanab et al. 2013, [62]	Jordan	243	6	90	10.9 [5.2]	54.9	12.75
B Banwat et al. 2016, [63]	Nigeria	130	–	80.8	4.9	65.8	0.1
Dayom DW et al. 2014, [25]	Nigeria	300	–	70	1.6	–	39
Deviprasad et al. 2016, [64]	India	114	2	38	1.62	9	9
Gitawati 2014, [65]	Indonesia	250	1	82	4.9	69.2	–
Gupta et al. 2011, [66]	India	97	6	94.8	12.2	–	26
Jassim 2010, [3]	Iraq	300	2007–2008	94	14.26	55.2	13.36
Justin et al. 2002, [67]	Tanzania	400	–	73.3	1.9	35.3	–
Kumar et al. 2013, [68]	India	500	6	74.6	4	28.5	1.56
Kusturica et al. 2012, [61]	Serbia	108	6	–	11.3	16.7	10.3
Kusturica et al. 2016, [69]	Serbia	383	over 8	–	11.4	–	9.2
Martin s et al. 2017, [70]	Brazil	267	2	–	3.7 [2.1]	38.4	5.7
Mirza et al. 2016, [28]	India	400	2012–2014	93.75	5.11 [3.42]	38	3.39
Ocan et al. 2014, [23]	Uganda	892	2	35.1	6 [5]	51.8	–
Ristic et al. 2016, [71]	Serbia	2600	6	–	8.2	56.3	12.22
Sooksriwong et al. 2013, [72]	Thailand	500	3	71	6.2	34	3.7
Teni et al. 2017, [73]	Ethiopia	771	1	44.2	1.85 [1]	41.1	3.14
Yousif et al. 2002, [74]	Sudan	469	2	97.7	4.4	47.2	18.2
Zargarzadeh et al. 2005, [75]	Iran	533	6	96	22.99 [20.1]	53.8	38.8

Dashes indicate information unavailable

### Risk of bias assessment

No articles were excluded based on quality appraisal. All the included studies acquired more than 60% of overall score. So that 55% (*n* = 11) of the studies were in the third quarter Q3 (≥75% of overall score). Most studies adopted an appropriate sampling method as random sampling was used much more often (75%, *n* = 15) than nonrandom sampling (25%, *n* = 5). Most studies had a good congruity between the research methodology and their research question, data collection, data analysis, and interpretation of results, so it can be said that most studies had good quality. The objectives were clearly stated, and in most studies, the results were well-reported (95%, *n* = 19). Of note, rate of wastage was explicitly reported in only three studies. However, for the remained ones, the required data were extracted according to the method firstly introduced by Zargarzadeh et al. [75] (Additional file 3).

### Prevalence of medicine storage

**Fig. 2:**
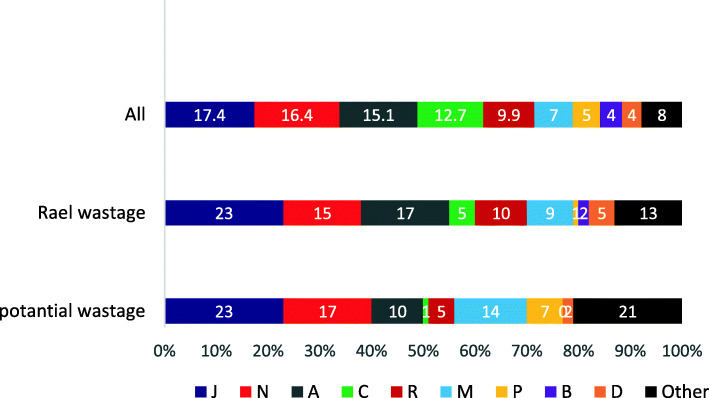
Rank of stored and wasted medicines by ATC category (average %)

**Fig. 3:**
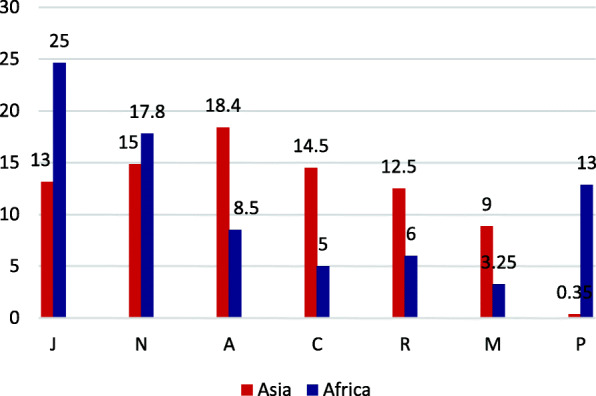
Comparison of stored medicines in Asia and Africa (average %)

According to the findings (Table 1), the prevalence of medicine storage ranged from 35.1% [23] to about 100% [16]. In addition, 13 studies reported more than 70% prevalence rate, which in half of them, it was at least 90%. Tablets were the most common form (over 60%) of medicines stored at home [23, 25, 28, 63, 64]. While syrup/ suspensions and capsule were ranked as the second and third ranks, respectively [25, 28, 63, 64, 74]. Only six studies had classified their results by ATC and for the remained studies, we classified the reported products in terms of the WHO’s guideline (See Additional file 4). The most widely stored medicine was Anti-infective for systemic use (J;17.4 ± 10.7%), followed by Nervous system (category N; 16.45 ± 6.6%), Alimentary tract drug (A; 15.1 ± 9.4%), Cardiovascular system (C; 12.7 ± 13.27%), Respiratory system (R; 9.97 ± 5.7%), Musculoskeletal system (M; 7.4 ± 5.7%), Antiparasitic products, insecticides and repellents (P; 5.3 ± 7.8%), Blood and blood forming organs (B; 4.25 ± 3.9%), and Dermatological agents (D; 3.85 ± 2.9%) (Fig. 2). The way of achieving the rank of the classes is given in Additional file 5. Frequency of individual’s medicines was reported in 3 studies consisting total of 1906 households [23, 62, 73]. The most common individual medicine product was Paracetamol (N; 10% ± 1.7%). The mean number of medicines stored per household varied from 1.6 [25] to 23 [75] (Table 1). In Africa, both the anti-infective and anti-malaria medicines were found as the most common therapeutic class of medication storage. However, in Asia, both the gastrointestinal system and cardiovascular medicines were the most common therapeutic classes of stored medicines (Fig. 3).

### Types and rate of wasted medicines

Out of 20 studies, only 17 studies [3, 16, 25, 28, 61–64, 66, 68–75] reported real and 17 studies [3, 16, 23, 28, 61–65, 67, 68, 70–75] reported potential rate of medicine wastage. According to the results, the potential rate of wastage was reported to be between 4.26 [16] and 69.2% [65], and the real rate of wastage was between 0.1 [63] and 38.8% [75] (Table 1). The way of calculating the potential waste for different studies is given in Additional file 6. Notably, anti-infective medicines (category J) had the first rank among both wastage types (Fig. 2) (See Additional file 5).

### Factors associated with medicine storage and wastage

Five studies [23, 62, 64, 73, 75] involving 2553 households performed statistical analysis; however, a limited number of them reported their results by odds ratios (Table 2). The commonly reported factors associated with medicine storage were as follows: income [23, 62, 73, 75], educational level [62, 75], age [64], family size [62], the presence of chronic illness [73, 75], female genders [23, 64], past successful treatment [23], working members [62], the presence of healthcare worker in the household [62, 64], and insurance coverage [75] (Table 2). The association between medicine wastage and patient-related factors was also analyzed by 4 studies [3, 16, 25, 69], highlighting that family size [25, 69], children aged under 12 years old [69], educational level [3, 25], unemployment [25], and per capita medical expenditure [16] were significantly associated with medicine wastage (Table 3).

**Table 2:** Factors associated with medicine storage

Variables	Study	Association (*P*-value)	Odds ratio	
			UOR (95%CI)	AOR (95% CI)
Income	Teni et al. 2017, [73]	↑ (< 0.05)		2.518 [1.215–5.221]
	Ocan et al. 2014, [23]	↑	1.76 [1.19–2.61]	
	Zargarzadeh et al. 2005, [75]	↑ (0.002)		
	Abushanab et al. 2013, [62]	↑ (0.034)		
Insurance coverage	Zargarzadeh et al. 2005, [75]	↑ (0.002)		
	Abushanab et al. 2013, [62]	NS		
Family size	Abushanab et al. 2013, [62]	↑ (0.004)		
	Zargarzadeh et al. 2005, [75]	NS		
Presence of chronic illness	Zargarzadeh et al. 2005, [75]	↑ (< 0.001)		
	Teni et al. 2017, [73]	↑ (< 0.05)		14.824 [9.072–24.2]
	Abushanab et al. 2013, [62]	NS		
Female gender	Ocan et al. 2014, [23]	↑	0.63 [0.5–0.9]	
	Deviprasad et al. 2016, [64]	↑ (0.01)		0.22 [0.07–0.7]
Past successful treatment	Ocan et al. 2014, [23]	↑	1.3[0.95–1.77]	
Working members	Abushanab et al. 2013, [62]	↑ (0.003)		
Education	Abushanab et al. 2013, [62]	↑ (< 0.001)		
	Zargarzadeh et al. 2005, [75]	↑ (0.003)		
	Teni et al. 2017, [73]	NS		
Occupation	Abushanab et al. 2013, [62]	NS		
	Zargarzadeh et al. 2005, [75]	NS		
Presence of healthcare worker	Teni et al. 2017, [73]	NS		
	Deviprasad et al. 2016, [64]	↑ (0.01)		7.22[1.52–34.21]
	Abushanab et al. 2013, [62]	↑		
	Zargarzadeh et al. 2005, [75]	↓ (< 0.001)		
Age	Deviprasad et al. 2016, [64]	↑ (0.03)		0.24 [0.06–0.89]
Children ≤6 years	Zargarzadeh et al. 2005, [75]	NS		
	Abushanab et al. 2013, [62]	NS		
	Deviprasad et al. 2016, [64]	NS		
Elderly ≥65 years	Zargarzadeh et al. 2005, [75]	NS		
	Abushanab et al. 2013, [62]	NS		
	Deviprasad et al. 2016, [64]	NS		

*UOR* Crude Odds ratio, *AOR* adjusted odds ratio

**Table 3:** Factors associated with medicine wastage

Variables	Study	Association (***P***-value)
**Family size**	Kusturica et al. 2016, [69]	↑ (0.002)
	Dayom DW et al. 2014, [25]	↑ (NR)
**Children ≤ 12 years**	Kusturica et al. 2016, [69]	↑ (0.019)
**Education**	Kusturica et al. 2016, [69]	↑ (0.007)
	Jassim 2010, [3]	↓ (*p* < 0.01)
	Dayom DW et al. 2014, [25]	↓ (NR)
**Unemployed**	Kusturica et al. 2016, [69]	Ns (0.08)
	Dayom DW et al. 2014, [25]	↑ (NR)
**Elderly ≥ 65 years**	Kusturica et al. 2016, [69]	Ns (0.09)
**household per capita medical expenditure**	Abou-Auda 2002, [16]	↓ (*p* < 0.001)

*NR* Not reported

### Cost

Expressed in terms of year 2011 US dollars, the estimates of the average cost of stored medicine per household in the 5 published studies included in this review, ranged more than 16-fold. Abushanab’s 2013 study reported the highest: US$ 100 [62], Gupta’s 2011 study reported the lowest estimate: US$ 6 [66]. The monetary value of wasted medicines accounted for 10 to 45% of households spending on medicines (Table 4).

**Table 4:** Cost of medicines (total and wasted)

Study	Total cost of medicines, ($)	Total cost of medicines per household, US $	Wastage medicines based on total cost (%)
Jordan [62]	21,900	100	24.4^b^
Saudi Arabia [16]	52,525	33.8	19.2^b^
Gulf countries [16]	2366	27.2^a^	25^b^
Thailand [72]	8925	25	9^c^
Iran [75]	3430	6.7	45^d^
India [66]	582	6	26^d^

^a^Average for Kuwait, United Arab Emirates, Qatar, and Oman combined

^b^ All wastage

^c^ potential wastage

^d^ Real wastage

### Storage conditions

**Fig. 4:**
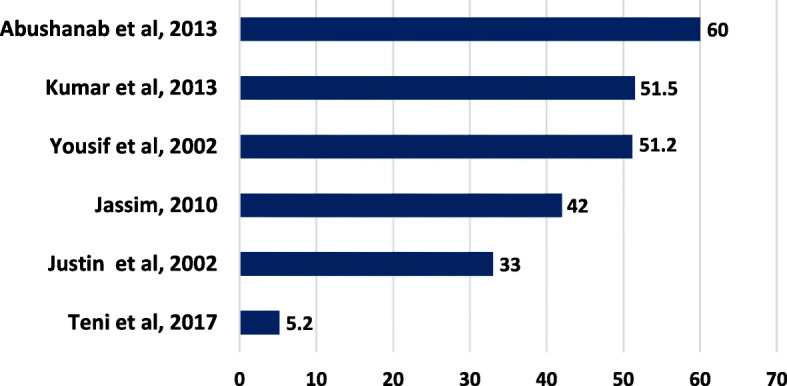
Storage conditions of medicine products found in households (% medicine)

According to WHO guidance on good storage practice, the appropriate storage condition is keeping the medicines in a clean and dry place, maintained within acceptable temperature limits, and out of the reach of children [76]. Storage condition was addressed in six studies involving 2683 households [3, 62, 67, 68, 73, 74]. In these studies, places such as medicine cabinet [62, 67, 68], refrigerator [3, 62, 67, 68, 73, 74], exposed to ventilation [3, 74], and First Aid box [67] were mentioned as appropriate places for storage of medicine. Appropriate storage rate was widely varied with some studies reporting as low as 5.2% in Ethiopia [73] to as high as 60% in Jordan [62] (Fig.4). Some other places such as kitchen, bathroom, bedroom, living room, cupboard, drawer, table, purse, and pockets on cloths were of most use places, which are considered as unsuitable places to store medicine.

### The disposal method of medicine

**Fig. 5:**
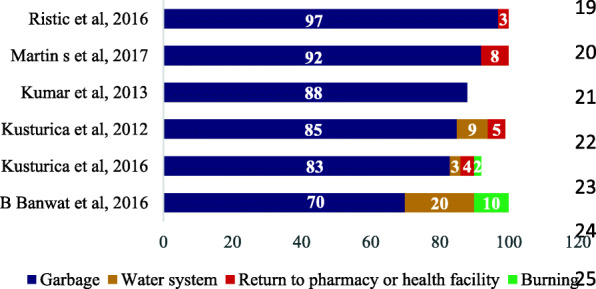
Disposal method of medicine (% household)

Of the 20 studies, 6 [61, 63, 68–71] articles addressed the disposal practice among households. Disposing medicines in the garbage was reported by 100% of the studies; however, this rate was varied from 70% in Nigeria [63] to 97% in Serbia [71]. It was shown that returning medicines to pharmacy or health center is the least practiced method of disposal among households. So, in India and Nigeria, none of the households used this method [63, 68]. In countries where the households adhered medicine take-back programs, this rate is still as so low as 3 to 8% (Fig. 5).

### Pooled estimation of the prevalence of stored and wasted medicines

#### Stored medicine

**Fig. 6:**
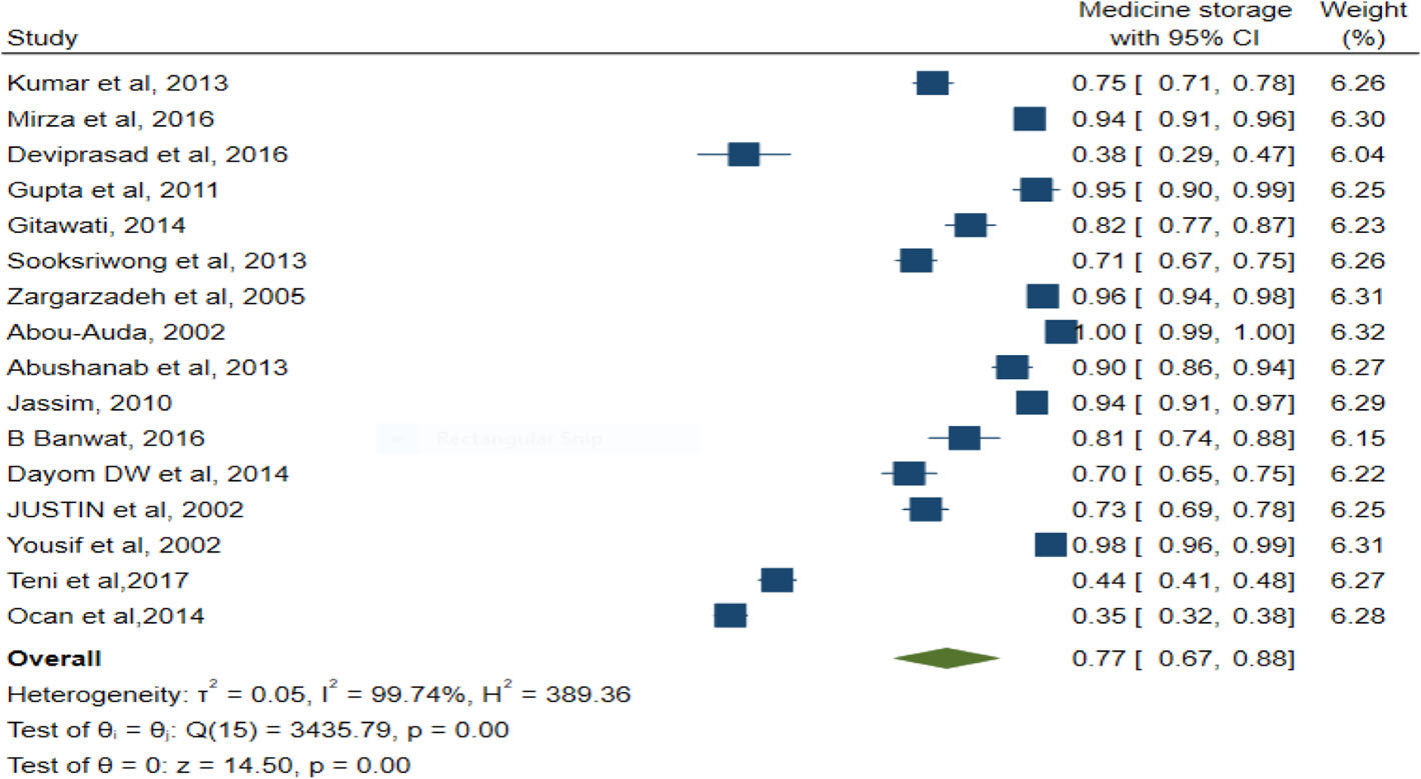
Forest plot assessing the prevalence of medicine storage among households, using data from 16 studies

Of 20 studies, 16 articles reported prevalence rate for medicine stored among households, which was considered for quantitative synthesis. The prevalence of medicine storage among households was 77% (95% CI; 67–88) with high heterogeneity between 16 studies (I^2^ = 99.74%; *p* = 0.00; Q = 3435.79) (Fig. 6).

#### Subgroup analyses among geographical regions

Subgroup analysis that was performed according to the United Nation’s geoscheme, showed that Southwest Asia had the highest rate of medicine storage as 95% (95% CI; 0.91–0.99, I^2^ = 95.22%; *p* = 0.00; Q = 58.24), followed by South and Southeast Asia as 76% (95% CI; 0.60–0.92, I^2^ = 98.99%; *p* = 0.00; Q = 249.5) and Sub-Saharan Africa as 67%(95% CI; 0.48–0.86, I^2^ = 99.48%; *p* = 0.00; Q = 1844.95) (See Additional file 7).

### The prevalence of real wastage

**Fig. 7:**
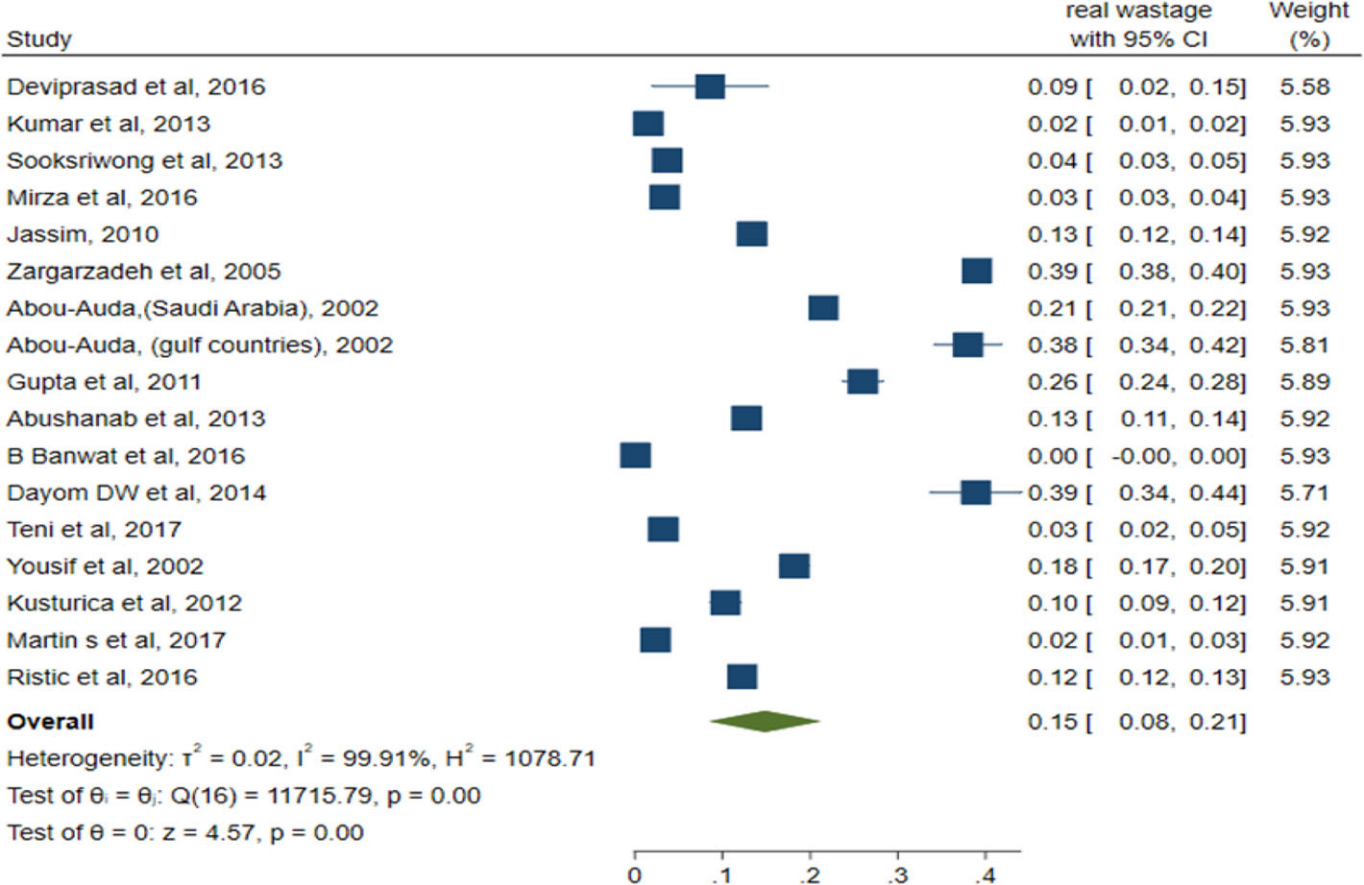
Forest plot assessing the prevalence of medicine real wastage among households, using data from 16 studies

Pooled prevalence of real medicine wastage for 16 studies across the globe was 15% (95% CI: 0.08–0.21) and still with high heterogeneity (I^2^ = 99.91%; *p* = 0.00; Q = 11,715.79), which was performed using random effect meta-analysis, as shown in Fig.7.

### Subgroup analyses based on geographical regions

The prevalence of real waste was 25% (95% CI; 0.14–0.36) in Southwest Asia (I^2^ = 99.82%; *p* = 0.00; Q = 1898.72) and 9% (95% CI: 0.00–0.18) and 15% (95% CI: − 0.02- 0.32) for South and Southeast Asia and Sub-Saharan Africa, respectively (See Additional file 7).

## Discussion

This systematic review and meta-analysis included 20 published studies conducted on a total of 10,898 urban households across 18 countries. The majority of the articles were from Asia and Africa, three from Europe, and one from South America. The pooled prevalence rates obtained from the included studies was 77% (95% CI; 67–88) for medicine storage and 15% (95% CI: 0.08–0.21) for real wastage, suggesting most of the urban households worldwide have unnecessary home medicine storage.

In our systematic review, the paucity of studies from Western Europe and American countries should not lead to underestimate the problem in that region or it means that medicine storage and wastage are not relevant to them. Moreover, many studies have recently been published indicating that a significant amount of medicines remained unused in European and American communities where some parts of them were returned to pharmacies or health facilities and the rest of them were inappropriately disposed [77–80]. We have not included these studies because of differences in their data collection method, while we believe that investigating their results would clarify many challenges in their societies.

Notably, 77% of households worldwide had storage, which was the highest rate among Southwest Asia (95%) and the lowest one in Sub-Saharan Africa (67%). Despite that the high prevalence among countries were explained by disease pattern and the associated health problems such as a high proportion Gastrointestinal tract medicines in Jordan [62]; malaria and anti-infective medicines in Tanzania [67], Iran [75], Ethiopia [73], Uganda [23], and Nigeria [25]; and cardiovascular and diabetes medicines in Palestine [24], India [68], and Mexico [81]. However, in most of the countries, the medicine storage and wastage were due to inappropriate prescription practice and inadequate patient adherence to treatment [3, 16, 23, 24, 27, 69, 73, 82]. Patient attitudes and social values were mentioned as underlying factors stimulating medicine storage and wastage in different settings. According to the studies performed in Saudi Arabia, Kuwait, UAE, Qatar, Oman, and India, patients gratify drug prescription as the outcome of their visit to public health facilities [16, 68]. The role of patient’s attitudes has been confirmed in studies by Hu et al. and Norris et al., where immigrants from developing countries such as China, India, Korea, and Egypt had different perceptions towards accessing and use of medicines compared to European and American counterparts [83, 84]. Gedif et al. in their study attributed the lower prevalence rate of medicine storage among Ethiopian households to the community’s confidence on traditional medicine relative to pharmaceutical products [45].

This review also found some evidences confirming that expired medicines are being used by households in low- and middle-income countries [25, 69]. Dayom et al. reported that more than 97% of expired medicine items found in households are either being used or are kept for future use [25]. As well, the same evidence has been reported from high-income countries [30, 85]; confirming that Australian households believe that the medicines could be used for either 1 year or longer after their expiration [85]. These findings highlighted the need for efforts on public education regarding the rational use of medicines, particularly with respect to medicine expiry date, in order to prevent the problems linked to public health [86].

Based on the literature, to some extent medicine wastage is inevitable because of several reasons such as patient death, treatment failure, medicine change, and side effects [7]. Then, in different studies solutions such as producing medicines in smaller packages as well as prescription of medicines for shorter time lags were endorsed as effective approaches [3, 67, 73, 75, 80, 87, 88]. Ekedahl et al. in their study proposed a feasible way to decrease the volume of unused medicines in which a small “starter pack” is prescribed whenever a new treatment is initiated. Of note, if the consumption stopped, only small volumes would be stocked or discarded [26]. Additionally, a recent study by Bach et al. reported 27 to 30% wastage in 3.5 mg vials of bortezomib compared to 1.5 mg vials [88].

The first and most stored medicines were group J (17.4%), among which, Antibiotics are the most known medicines. Despite the fact that in many health systems antibiotics were authorized as prescription-only drugs, they are easily purchased with no prescription [3, 16, 23, 28, 65, 75]. The gap between the legislation and daily practice, over expectation about antibiotics effectiveness, low awareness regard to antibiotic resistance, adverse event, and high cost of physician consultation have been cited as reasons for antibiotics storage [89–93]. The same evidence has been reported by Sawair et al., indicating that patients visited the second physician because they did not receive any antibiotic from the first physician [94]. Kelly et al. in their study have cited microbial resistance as a significant public health concern and highlighted that one way of tackling microbial resistance is to limit both the prescription and use of anti-infective medicine for non-bacterial infections especially in societies with lower health literacy level [85].

Paracetamol (Acetaminophen) belonging to group N, was the most stored medicine by households. This finding is unsurprising, because the paracetamol is the first-line treatment for pain and fever management [1, 24, 62, 63]. However, the availability, affordability, convenience, marketing, and misconception about its safety compared to NSAIDs (Non-steroidal anti-inflammatory drugs) has made the paracetamol family the first choice of self-medication among people [1, 24, 62, 63, 65]. Although a few studies reported that keeping a limited stock of these medicines might be cost-saving due to lower physician visit [3, 28, 65, 66] more studies warned about the rising rate of self-medication as well as medicine wastage and its undesirable consequences [28, 95–97].

According to the results of analysis reported by 6 studies covering 4580 households, medicine storage and wastage were found to be positively associated with households purchasing power; indicating that in households with higher income or in countries whose medicine price is lower, both storage and wastage are more prevalent. This finding has also been confirmed by studies from high-income countries reporting that this problem exists in all over the world and there are factors beyond real health needs that are associated with demand and utilization medicines [30, 79, 98, 99].

Insurance coverage was recognized as a positive motive for more storage and wastage of medicines among households [75]. This finding suggested that, although having medical insurance could financially protect household members against catastrophic health expenditures, the lack of strict control on prescription, selling, and demand of medicine could encounter health systems to the increased cost of unnecessary storage and wastage of medicines. Moreover, Sweileh and Zargarzadeh in their study reported a stronger correlation between having medical insurance and medicine storage [24, 75].

The impact of education on drug storage is interestingly different from that on wastage. Most of the literature reported a positive association between the level of education of the household’s head and the amount of medicine storage [3, 24, 62, 74, 75]. However, the number of medicine wastage was reported as higher for illiterate or less educated counterparts [3, 25, 74]. Accordingly, it is obvious that education level could prevent drug wastage through better compliance with the treatment or better storage practice [3, 23, 28, 62, 74]. Nevertheless, according to the previous studies, educated people more tend to purchase more medicines from retail pharmacies and store medicines for future use, so they are more prone to self-medication [23, 28, 62, 74].

Some significant differences were found between men and women in terms of medicine storage modality, amount, and wastage. Notably, the higher the rate of in-home storage among women [23, 64], the lower the wastage rate linked to households who stored medicines and were organized by female subjects [70]. Other studies have confirmed high storage rates among women [70, 99, 100] by stating some reasons such as gender-related physiopathology, lifestyle, contact to the health system, and other biological differences like pregnancy [100, 101]. However, other variations such as women’s tendency to excessive purchase and self-medication, lacks medical reasoning, so they can potentially be considered as the focal point for improvement [102]. A study by Becker showed that wasted medicine was more frequently used by men compared to women [87].

Considering our findings, there was a considerable variation in medicine storage place among households in all geographic regions. Since the storage location was reported to be associated with the decreased adherence to treatment, unfavorable clinical outcomes and potentially hazardous consequences [103–105], WHO guideline [76] seems to be a good solution to solve this problem in which the appropriate place is keeping medicine in an enclosed and cool or dry place, which is inaccessible for children.

The majority of the studies (75%) highlighted that most countries have no guidelines for disposal of medicines, which not only imposes a financial burden on society, but also creates environmental hazards through the accumulation of chemical ingredients in landfills and freshwater resources. In most developed health systems, several schemes have been developed for the disposal of unused medicines that could be adopted as a practical solution by low and middle-income countries. For example, the National Return and Disposal of Unwanted Medicines (Nat RUM) scheme in Australia can be named that provides a route to return leftover medicines to community pharmacies [77]. Studies conducted in Sweden and Australia suggested a unitary medicine take-over system or educational campaigns at the national level instead of state-run programs [77, 106]. In Nebraska, especial boxes are placed in pharmacies that allow consumers to return their excessive medicines to the pharmacy [107]. Arkaravichien et al. [108] and Yang et al. [109] in their studies proposed that the national health system can provide financial incentives for pharmacies and households participating in take-back schemes.

### Strengths and limitation of the study

This systematic review focused on the most important part of the consumption chain, which is before medicine wastage as well as its related harms to the environment. Furthermore, most of the home storages were reviewed and then reported, so we believe that our study’s results can provide evidence for decision-making at both individual and community levels. Since all the included studies have focused on quantitative analysis of medicine storage, so the household’s preferences or their experiences as one of the key influential factors on establishing inappropriate storage and wastage, has not been addressed decently. Therefore, future research investigating this gap seems to be beneficial.

## Conclusion

This systematic review and meta-analysis gathered evidences from different geographical and demographical settings, implying that in-home medicine storage is a worldwide problem that could consequently lead to irrational use, wastage of medicine, and the associated hazardous outcomes. The prevalence rates of medicine storage and wastage are high among households worldwide and higher in regions where lacks inappropriate regulation and control on prescription and utilization of medicine. So, it is necessary to stress that factors beyond medical needs are associated with medicine storage among households, which urges the administration of effective strategies related to the supply and demand side of the medicine consumption chain along with proposing appropriate public health strategies such as increasing public knowledge on risks of storing medicines. The first necessary step to mitigate home storage is establishing an adequate legislation and strict enforcement of regulation on dispensing, prescription, and marketing of medicines. Furthermore, proper educational interventions targeting both health professionals and community members could be helpful in this regard. Patients’ pressure on excessive prescription, irrational storage, and use of medicines and inappropriate disposal practices deserve efficient community-centered programs for increasing awareness on these issues. The current data suggest that in low- and middle-income countries, health systems should necessarily pay more attention to setting medicine return back programs, in order to prevent environmental hazardous effects of improper disposal of wasted medications.

All the data generated or analyzed during this study are included in this published article (and in its supplementary information files).

## Supplementary Information

**Additional file 1.** PRISMA 2009 Checklist.

**Additional file 2.** Search strategy in PubMed.

**Additional file 3.** Critical appraisal results of the included studies.

**Additional file 4.** Pharmaceutical groups according to ATC classification.

**Additional file 5. **Ranking of medicine product according to the ATC system (wasted and total medicines).

**Additional file 6.** Dividing potential waste into constituent variables.

**Additional file 7. **Forest plot assessing the prevalence of medicine storage and real wastage.

## Data Availability

All data generated or analyzed during this study are included in this published article (and its supplementary information files).

## Abbreviations

WHO: World Health Organization
ATC: Anatomical Therapeutic Chemical
DDD: Daily Dose Defined
N: Nervous system
J: Anti-infective for systemic use
A: Alimentary tract drug
B: Blood and blood forming organs
C: Cardiovascular system
R: Respiratory system
M: Musculoskeletal system
P: Antiparasitic products, insecticides and repellents
D: Dermatological agents
UOR: Unadjusted odds ratio
AOR: Adjusted odds ratio
GIT: Gastrointestinal tract
NSAIDs: Non-steroidal anti-inflammatory drugs.
