# Supplementary material for: Medicine storage, wastage, and associated determinants among urban households: a systematic review and meta-analysis of household surveys
Source: BMC Public Health. 2021 Jun 12;21:1127. doi: 10.1186/s12889-021-11100-4 (PMC8196539; doi:10.1186/s12889-021-11100-4)
Supplement: Supplementary file 2 — Additional file 2. Search strategy in PubMed. [file 12889_2021_11100_MOESM2_ESM.docx]

**Additional file 2**: Search strategy in PubMed

(Medic* wast*[Title/Abstract] OR unwanted medic*[Title/Abstract] OR unwanted pharm*[Title/Abstract] OR unwanted drug*[Title/Abstract] OR unused medic*[Title/Abstract] OR unused pharm*[Title/Abstract] OR unused drug*[Title/Abstract] OR drug* wast*[Title/Abstract] OR pharm* wast*[Title/Abstract] OR prescription* wast*[Title/Abstract] OR unnecessary medic*[Title/Abstract] OR unnecessary drug*[Title/Abstract] OR unnecessary pharm*[Title/Abstract] OR extra medic*[Title/Abstract] OR extra drug*[Title/Abstract] OR extra pharm*[Title/Abstract] OR surplus medic*[Title/Abstract] OR surplus drug*[Title/Abstract] OR surplus pharm*[Title/Abstract] OR untouched medic*[Title/Abstract] OR untouched drug*[Title/Abstract] OR untouched pharm*[Title/Abstract] OR remain* medic*[Title/Abstract] OR remaining drug*[Title/Abstract] OR remaining pharm*[Title/Abstract] OR returned medic*[Title/Abstract] OR returned drug*[Title/Abstract] OR returned pharm*[Title/Abstract] OR leftover drug*[Title/Abstract] OR leftover medic*[Title/Abstract] OR leftover pharm*[Title/Abstract] OR expire* drug*[Title/Abstract] OR expire* pharm*[Title/Abstract] OR expire* medic*[Title/Abstract] OR drug* stock*[Title/Abstract] OR pharm* stock*[Title/Abstract] OR medic* stock*[Title/Abstract] OR reused medic*[Title/Abstract] OR reused pharm*[Title/Abstract] OR reused drug*[Title/Abstract]) AND (home storage[Title/Abstract] OR In-home storage[Title/Abstract] OR household*[Title/Abstract])
