# Supplementary material for: Medicine storage, wastage, and associated determinants among urban households: a systematic review and meta-analysis of household surveys
Source: BMC Public Health. 2021 Jun 12;21:1127. doi: 10.1186/s12889-021-11100-4 (PMC8196539; doi:10.1186/s12889-021-11100-4)
Supplement: Supplementary file 3 — Additional file 3. Critical appraisal results of the included studies. [file 12889_2021_11100_MOESM3_ESM.docx]

**Additional file 3: Critical appraisal results of the included studies**

| Citation | Q1 | Q2 | Q3 | Q4 | Q5 | Q6 | Q7 | Q8 | Q9 | Q10 | Q11 | Q12 | Q13 | Q14 | Q15 | Q16 | Q17 | Total score |
| --- | --- | --- | --- | --- | --- | --- | --- | --- | --- | --- | --- | --- | --- | --- | --- | --- | --- | --- |
| Abou-Aida 2002,[16] | Y | Y | U | Y | Y | Y | P | Y | Y | Y | Y | Y | Y | Y | Y | Y | Y | 0.91 |
| Abushanab et al 2013,[67] | Y | Y | Y | Y | Y | P | Y | Y | Y | Y | Y | Y | Y | Y | Y | Y | Y | 0.97 |
| B Banwat et al 2016, [63] | Y | N | U | Y | P | Y | P | P | Y | P | P | Y | Y | Y | Y | Y | Y | 0.73 |
| Dayom DW et al 2014, [25] | Y | N | U | Y | Y | Y | Y | P | P | Y | Y | U | P | Y | P | Y | Y | 0.7 |
| Deviprasad et al 2016, [64] | Y | N | Y | Y | Y | P | N | Y | Y | Y | Y | Y | Y | Y | Y | Y | U | 0.79 |
| Gitawati 2014, [74] | Y | N | U | Y | P | Y | P | Y | N | Y | P | Y | Y | P | Y | Y | Y | 0.7 |
| Gupta et al 2011, [69] | Y | N | U | Y | Y | Y | N | Y | P | Y | P | Y | Y | U | p | Y | U | 0.62 |
| Jassim 2010, [3] | Y | N | U | Y | P | Y | P | Y | P | Y | N | U | Y | N | Y | Y | Y | 0.62 |
| JUSTIN et al 2002, [75] | Y | N | U | Y | P | Y | Y | P | Y | P | P | Y | Y | N | Y | Y | U | 0.65 |
| Kusturica et al,2012, [61] | Y | N | Y | Y | N | Y | N | Y | Y | P | Y | Y | Y | Y | Y | Y | Y | 0.79 |
| Kumar et al 2013, [70] | Y | N | N | Y | Y | Y | P | Y | Y | Y | P | Y | Y | Y | Y | Y | Y | 0.82 |
| Kusturica et al 2016, [71] | Y | N | Y | Y | P | Y | Y | Y | P | Y | Y | Y | Y | Y | P | Y | Y | 0.85 |
| Martin s et al 2017, [68] | Y | N | Y | Y | P | Y | Y | Y | P | Y | Y | Y | Y | Y | Y | P | Y | 0.85 |
| Mirza et al 2016, [28] | Y | N | Y | Y | Y | Y | Y | P | Y | P | N | Y | Y | Y | Y | Y | Y | 0.82 |
| Ocan et al 2014, [23] | Y | N | Y | Y | Y | P | Y | Y | Y | Y | Y | Y | Y | N | Y | Y | Y | 0.79 |
| Ristic et al 2016, [73] | Y | N | U | Y | Y | Y | Y | Y | P | P | P | Y | Y | N | Y | Y | U | 0.68 |
| Sooksriwong et al 2013, [72] | Y | N | U | Y | Y | Y | N | Y | Y | Y | N | N | Y | U | Y | Y | U | 0.59 |
| Teni et al 2017, [66] | Y | N | Y | Y | Y | Y | Y | Y | Y | Y | Y | Y | Y | Y | Y | Y | Y | 0.94 |
| Yousif et al 2002, [65] | Y | N | U | Y | Y | Y | N | Y | P | Y | Y | Y | Y | U | Y | Y | Y | 0.73 |
| Zargarzadeh et al 2005, [62] | Y | Y | Y | Y | Y | Y | Y | Y | Y | Y | Y | Y | Y | Y | Y | Y | Y | 1 |

Y, yes(2); P, partial(1); N, no(0); U, unclear(0); Na, Not applicable
 Q1: Did the study ask a clearly-focused and relevant question(s) (aims, objectives)?
 Q2: Was the definition of waste for this study clearly explained?
 Q3: Was ethics approval reported?
 Q4: Was the study design/method justified and appropriate for the research question(s)?
 Q5: Were limitations of study design/method considered?
 Q6: Was the sampling population and strategy clear and justified?
 Q7: Was the sample size justified?
 Q8: Was there appropriate statistical analysis?
 Q9: Were the data collection tools described, piloted and validated?

Q10: Were analysis strategies (quantitative and any open comments) clear and justified?
 Q11: Were participant characteristics sufficiently described?
 Q12: Is the loss of any of the participants explained?
 Q13: Are the results of the study clearly explained and do the results address the original research question?
 Q14: Were limitations (bias, confounders, generalisability etc) of findings considered?
 Q15: Were all important outcomes considered so the results can be applied?
 Q16: Are the conclusions supported by the findings?
 Q17: Is the study free of conflict of interest?
