# Supplementary material for: Medicine storage, wastage, and associated determinants among urban households: a systematic review and meta-analysis of household surveys
Source: BMC Public Health. 2021 Jun 12;21:1127. doi: 10.1186/s12889-021-11100-4 (PMC8196539; doi:10.1186/s12889-021-11100-4)
Supplement: Supplementary file 4 — Additional file 4. Pharmaceutical groups according to ATC classification. [file 12889_2021_11100_MOESM4_ESM.docx]

|  | | | | | | | | | | | | | |
| --- | --- | --- | --- | --- | --- | --- | --- | --- | --- | --- | --- | --- | --- |
| **Additional file 4:** Pharmaceutical groups according to ATC classification. | | | | | | | | | | | | | |
| **A** | Antispasmodics | | GIT drugs | | | | | Drugs for treatment of peptic ulcer | | | | | Antispasmodics |
|  | Gastrointestinal | | Nutrient Supplements | | | | | Spasmolytic | | | | | Vitamins and minerals |
|  | Anti-diabetic Drugs | | Antacids | | | | | Oral rehydration salt | | | | | Anti -diarrheal agents |
|  | Vitamins & minerals | | Antidiarrheal, laxatives, others | | | | | Antacids, antireflux agents and antiulcer agents | | | | | |
|  |  | | | | | | | | | | | | |
| **B** | Obstetrics, gynecology, and urinary tract disorders | | | | | | | Nutrition and blood | | | | | Antiplatelet |
|  |  | | | | | | | | | | | | |
| **C** | Cardiovascular | | Antihypertensive | | | | Antihypertensive drugs (including diuretics) | | | | | | |
|  |  | | | | | | | | | | | | |
| **D** | Antiulcer | Antifungals | | Skin | Topicals preparations | | | | Topical non-steroid products | | | | Topical steroid products |
|  |  | | | | | | | | | | | | |
| **H** | Endocrine system | | Corticosteroids | | | | Hormones, systemic (except oral contraceptives) | | | | | | Steroid Preparations |
|  |  | | | | | | | | | | | | |
| **J** | Anti-infective agents | | Antibacterial | | | Beta lactams | | Antiretroviral | | | Antibiotics | | Systemic antiviral |
|  |  | | | | | | | | | | | | |
| **L** | Cytotoxic | | Malignant disease and immunosuppression | | | | | | | | | | |
|  |  | | | | | | | | | | | | |
| **M** | NSAIDs | | | | | | | | | | | | |
|  |  | | | | | | | | | | | | |
| **N** | Central Nervous System (CNS) | | | | | | Analgesics | Antipyretic Analgesics | | | | Antipsychotics | Antidepressants |
|  | Non-opioid analgesics and antipyretic drugs | | | | | | | Opioid antitussives/analgesics | | | | | Benzodiazepines |
|  |  | | | | | | | | | | | | |
| **P** | Antimalarial | | Antihelmintic | | | | | Anti-parasitic | | | | | |
|  |  | | | | | | | | | | | | |
| **S** | Ear, nose, and oropharynx | | | | | | Eye | Eye/nose and skin agents | | | | | |
|  |  | | | | | | | | | | | | |
| **R** | Allergy and immune system | | | | | | Drugs used to treat asthma | | | Respiratory | | | Antihistamines |
|  | Cough mixtures | | Anti-allergic drugs (systemic) | | | | | | | | | | |
