# Supplementary material for: Medicine storage, wastage, and associated determinants among urban households: a systematic review and meta-analysis of household surveys
Source: BMC Public Health. 2021 Jun 12;21:1127. doi: 10.1186/s12889-021-11100-4 (PMC8196539; doi:10.1186/s12889-021-11100-4)
Supplement: Supplementary file 5 — Additional file 5. Ranking of medicine product according to the ATC system (wasted and total medicines). [file 12889_2021_11100_MOESM5_ESM.docx]

**[Additional](https://wiki.joannabriggs.org/display/MANUAL/Appendix+5.1%3A+Critical+Appraisal+Instrument+for+Studies+Reporting+Prevalence+Data)****[file 5:](https://wiki.joannabriggs.org/display/MANUAL/Appendix+5.1%3A+Critical+Appraisal+Instrument+for+Studies+Reporting+Prevalence+Data)**

1.Types of drug product found in households classiﬁed according to the ATC system (all storage).

| Medicine | Abushanab et al, 2013 [67] | Zargarzadeh et al ,2005 [62] | Ocan et al, 2014 [23] | Jassim, 2010 [3] | Abou-Auda, 2002 [16] | Abou-Auda, 2002 [16] | Justin et al, 2002 [75] | Martins et al, 2017 [68] | Teni et al, 2017 [66] | Yousif et al, 2002 [65] | B Banwat et al, 2016 [63] | Sooksriwong et al, 2013 [72] | Mirza et al, 2016 [28] | Ristic et al, 2016 [73] | Deviprasad et al , 2016 [64] | Average (%) |
| --- | --- | --- | --- | --- | --- | --- | --- | --- | --- | --- | --- | --- | --- | --- | --- | --- |
| N | 17.3 | 24 | 20 | 24 | 16 | 14 | 22 | 22 | 13 | 11 | 23 | 16 | 2 | 17 | 5.5 | **16.4** |
| J | 10.6 | 14 | 45 | 26 | 14 | 18 | 13 | - | 24 | 22 | 19 | - | 8 | 11 | 1.5 | **17.4** |
| A | 20.7 | 13 | 4.7 | 16 | 11 | 10 | - | 17 | 19 | 0 | 10 | 14 | 22 | 14 | 40.5 | **15.1** |
| C | 10.9 | 13 | 0 | 0 | 3.7 | 10 | 0 | 29 | 18 | - | 2 | - | 18 | 14 | 46 | **12.7** |
| R | 12 | 9.7 | 3.2 | 6.5 | 21 | 20 | 9.9 | - | 2.9 | - | 8 | 6 | 15 | 8.5 | 7 | **9.9** |
| M | 12.9 | 8.6 | 0 | 11 | 6.3 | 8 | 0 | - | 13 | - | 0 | 8 | 16 | 13 | 0 | **7.4** |
| P | 0.7 | 0 | 17 | 1.8 | 0 | 0 | 25 | 0 | 4.3 | 10 | 8 | - | 0 | 2.5 | 0 | **5** |
| B | 4.6 | 9.7 | 2.8 | 0 | 6.6 | 4 | 6 | - | 0.9 | 0 | 12 | - | 0 | 4 | 0 | **4** |
| D | 5.4 | 0 | 1.7 | 0 | 5.9 | 5 | 7.3 | - | 2.4 | - | - | - | 8 | 4 | 0 | **3.6** |

**2. Real wastage:**

| Medicine | Abushanab et al,2013 [67] | Jassim, 2010 [3] | Ristic et al,2016 [73] | Gupta et al, 2011 [69] | Dayom DW et al, 2014 [25] | Kusturica et al,2016 [71] | Average(%) |
| --- | --- | --- | --- | --- | --- | --- | --- |
| J | 6.8 | 37.4 | 16 | 15 | 43 | 17 | **23** |
| A | 25 | 3 | 15 | 29 | 14 | 14 | **17** |
| N | 12.2 | 25 | 9.6 | 24 | 7.5 | 11 | **15** |
| R | 18 | 3 | 8.4 | 12 | 5.2 | 11 | **10** |
| M | 13.4 | 14.5 | 13 | 0 | 3 | 12 | **9** |
| D | 10 | 0 | 6.5 | 0 | 0 | 16 | **5** |
| C | 5.5 | 0 | 8 | 0 | 12 | 6.7 | **5** |
| B | 0 | 0 | 3.6 | 0 | 5.2 | 3.7 | **2** |
| S | 4.5 | 0 | 0 | 0 | 0 | 4.5 | **1.5** |
| P | 0 | 0 | 0 | 0 | 5.2 | 0.2 | **1** |
| H | 0 | 3 | 0 | 0 | 4.5 | 0 | **1** |

**3.Potential waste.**

| Medicine | Ocan et al,2014[23] | Jassim, 2010 [3] | Kumar et al,2013 [70] | Ristic et al,2016 [73] | Average(%) |
| --- | --- | --- | --- | --- | --- |
| J | 41.4 | 28.1 | 19 | 3.5 | **23** |
| N | 16.7 | 26 | 0 | 25 | **17** |
| M | 0 | 10.6 | 25 | 20 | **14** |
| A | 5.8 | 16.5 | 18 | 0 | **10** |
| P | 22 | 2 | 0 | 5 | **7.3** |
| R | 3.3 | 5.5 | 0 | 10 | **5** |
| D | 1.9 | 0.5 | 0 | 7 | **2.4** |
| C | 0.1 | 0 | 0 | 4 | **1** |
