# Supplementary material for: Medicine storage, wastage, and associated determinants among urban households: a systematic review and meta-analysis of household surveys
Source: BMC Public Health. 2021 Jun 12;21:1127. doi: 10.1186/s12889-021-11100-4 (PMC8196539; doi:10.1186/s12889-021-11100-4)
Supplement: Supplementary file 6 — Additional file 6. Dividing potential waste into constituent variables. [file 12889_2021_11100_MOESM6_ESM.docx]

| **Potential Wastage (%)** | **Dividing potential waste into constituent variables** | | **Study** |  |
| --- | --- | --- | --- | --- |
| 4.26 | **-** | Unused | Abou-Auda, 2002 [16] | **1** |
| 54.9 | never used since dispensing(15.04) | Currently unused(39.9) | Abushanab et al, 2013 [67] | **2** |
| 65.8 | - | Unused | B Banwat et al, 2016 [63] | **3** |
| 9 | - | Not ongoing | Deviprasad et al, 2016 [64] | **4** |
| 69.2 | For future use(37.6) | Leftover(31.6) | Gitawati, 2014 [74] | **5** |
| 55.2 | For future use | Leftover | Jassim, 2010 [3] | **6** |
| 35.3 | - | Not in use | Justin et al, 2002 [75] | **7** |
| 16.7 | - | No longer in being used | Kusturica et al, 2012 [61] | **8** |
| 28.5 | - | Not used at all | Kumar et al, 2013 [70] | **9** |
| 38.4 | - | Not used | Martin s et al, 2017 [68] | **10** |
| 38 | For future use (18) | Leftover (20) | Mirza et al, 2016 [28] | **11** |
| 51.8 | For future use (20.1) | Leftover (31.7) | Ocan et al, 2014 [23] | **12** |
| 56.3 | - | If necessary | Ristic et al, 2016 [73] | **13** |
| 34 | - | Leftover | Sooksriwong et al, 2013 [72] | **14** |
| 41.1 | For future use (25.1) | Kept with no purpose(16) | Teni et al, 2017 [66] | **15** |
| 47.2 | For future use | - | Yousif et al, 2002 [65] | **16** |
| 53.8 | - | **N**ot in use | Zargarzadeh et al, 2005 [62] | **17** |

[**Additional** **file 6:**](https://wiki.joannabriggs.org/display/MANUAL/Appendix+5.1%3A+Critical+Appraisal+Instrument+for+Studies+Reporting+Prevalence+Data) Dividing potential waste into constituent variables.
