# Supplementary material for: Medicine storage, wastage, and associated determinants among urban households: a systematic review and meta-analysis of household surveys
Source: BMC Public Health. 2021 Jun 12;21:1127. doi: 10.1186/s12889-021-11100-4 (PMC8196539; doi:10.1186/s12889-021-11100-4)
Supplement: Supplementary file 7 — Additional file 7. Forest plot assessing the prevalence of medicine storage and real wastage. [file 12889_2021_11100_MOESM7_ESM.docx]

**Medicine storage:**


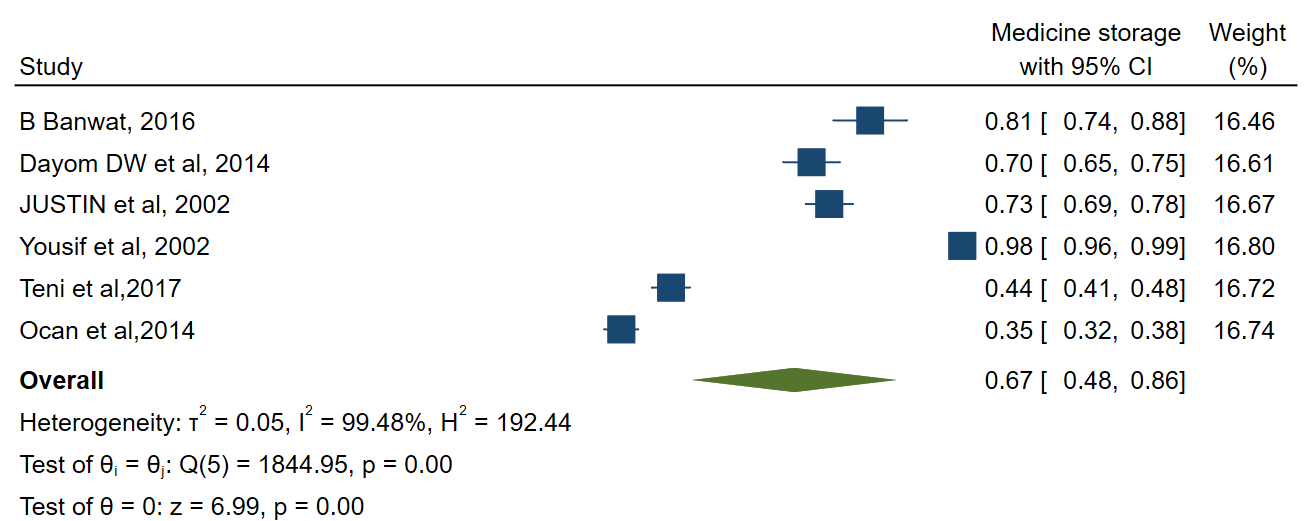


1. Forest plot assessing the prevalence of medicine storage among households, Sub-Saharan Africa


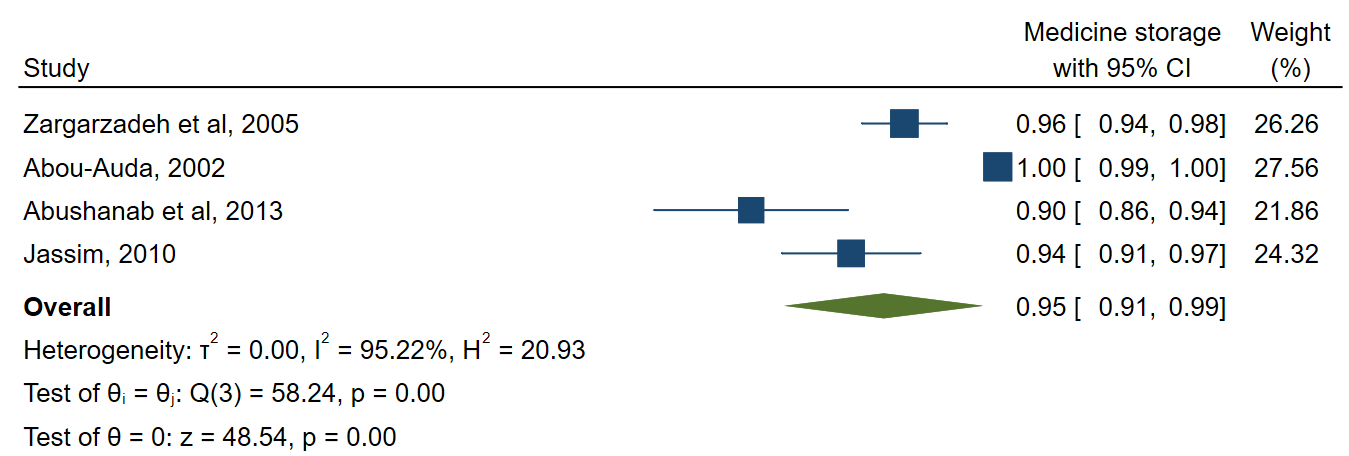


2.Forest plot assessing the prevalence of medicine storage among households, Southwest Asia


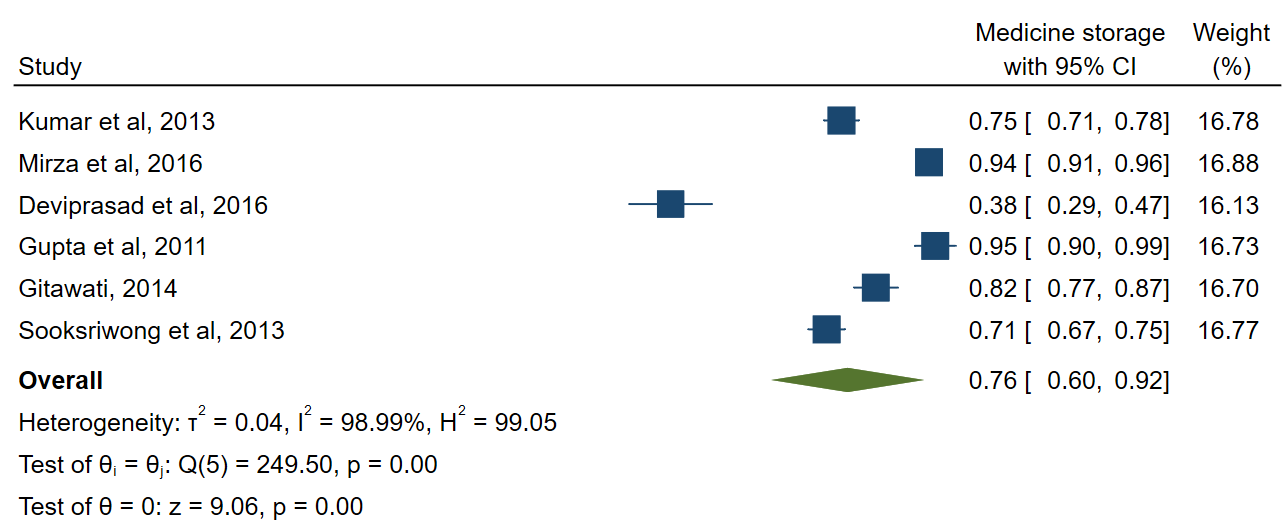


3.Forest plot assessing the prevalence of medicine storage among households, South and Southeast Asia

**Real wastage:**


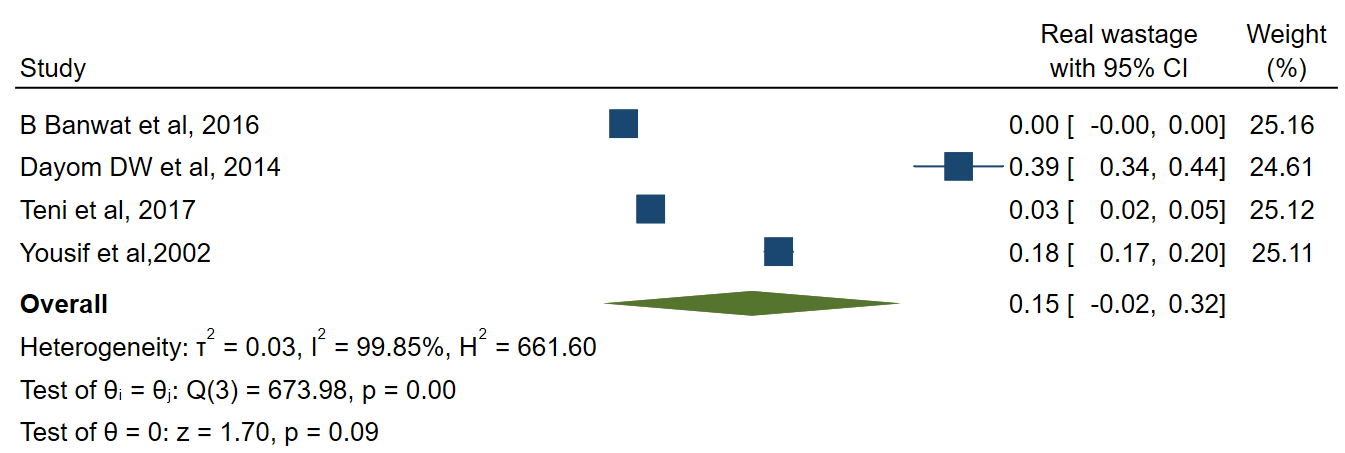


1. Forest plot assessing the prevalence of real wastage among households, Sub-Saharan Africa


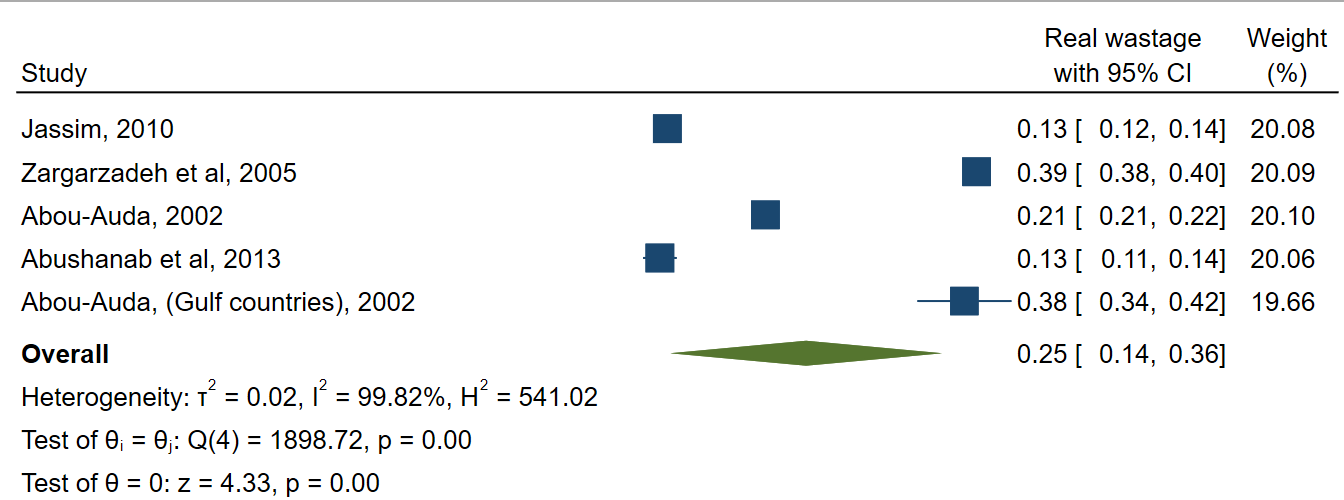


2. Forest plot assessing the prevalence of real wastage among households, Southwest Asia


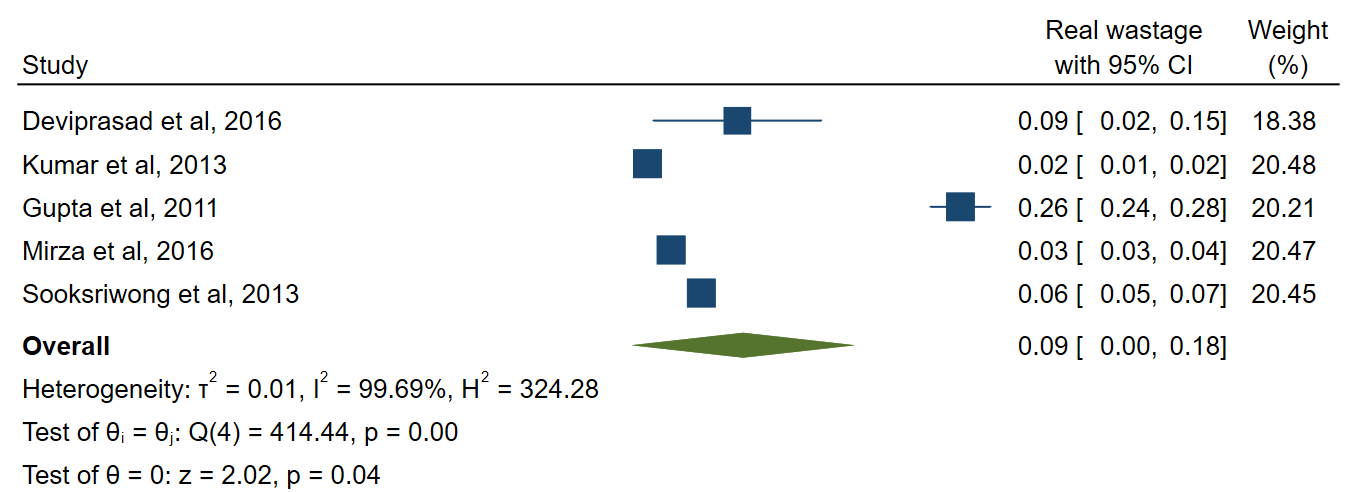


3. Forest plot assessing the prevalence of real wastage among households, South and Southeast Asia
